# Supplementary material for: Fluorescent Aerolysin (FLAER) Binding Is Abnormally Low in the Clonal Precursors of Acute Leukemias, with Binding Particularly Low or Absent in Acute Promyelocytic Leukemia
Source: Int J Mol Sci. 2024 Nov 5;25(22):11898. doi: 10.3390/ijms252211898 (PMC11593337; doi:10.3390/ijms252211898)
Supplement: Supplementary file 1 [file ijms-25-11898-s001.zip › ijms-3238593-supplementary.pptx]

## Slide 1
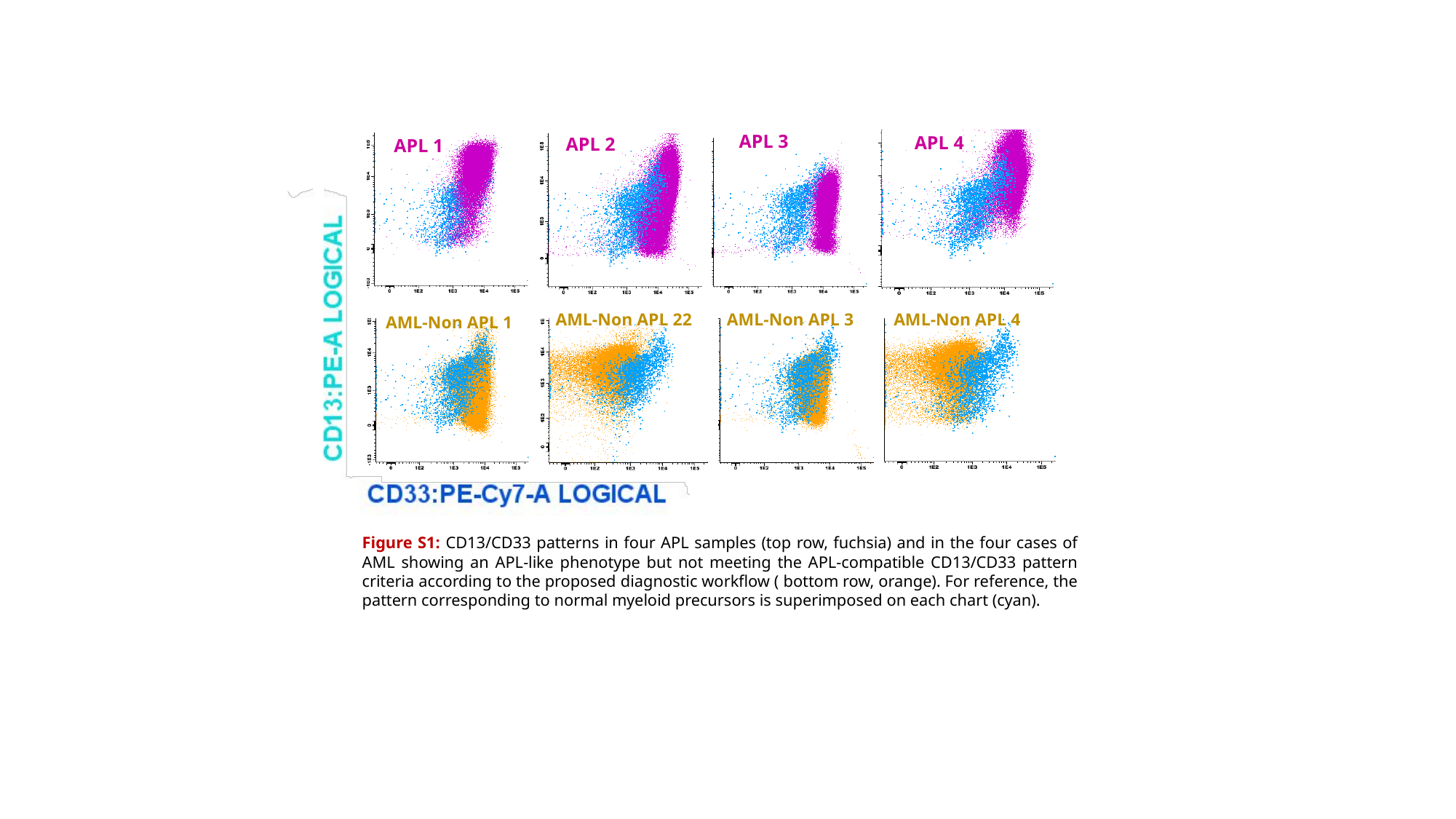

APL 3
APL 4
APL 2
APL 1
AML-Non APL 22
AML-Non APL 3
AML-Non APL 4
AML-Non APL 1
Figure S1: CD13/CD33 patterns in four APL samples (top row, fuchsia) and in the four cases of AML showing an APL-like phenotype but not meeting the APL-compatible CD13/CD33 pattern criteria according to the proposed diagnostic workflow ( bottom row, orange). For reference, the pattern corresponding to normal myeloid precursors is superimposed on each chart (cyan).

## Slide 2
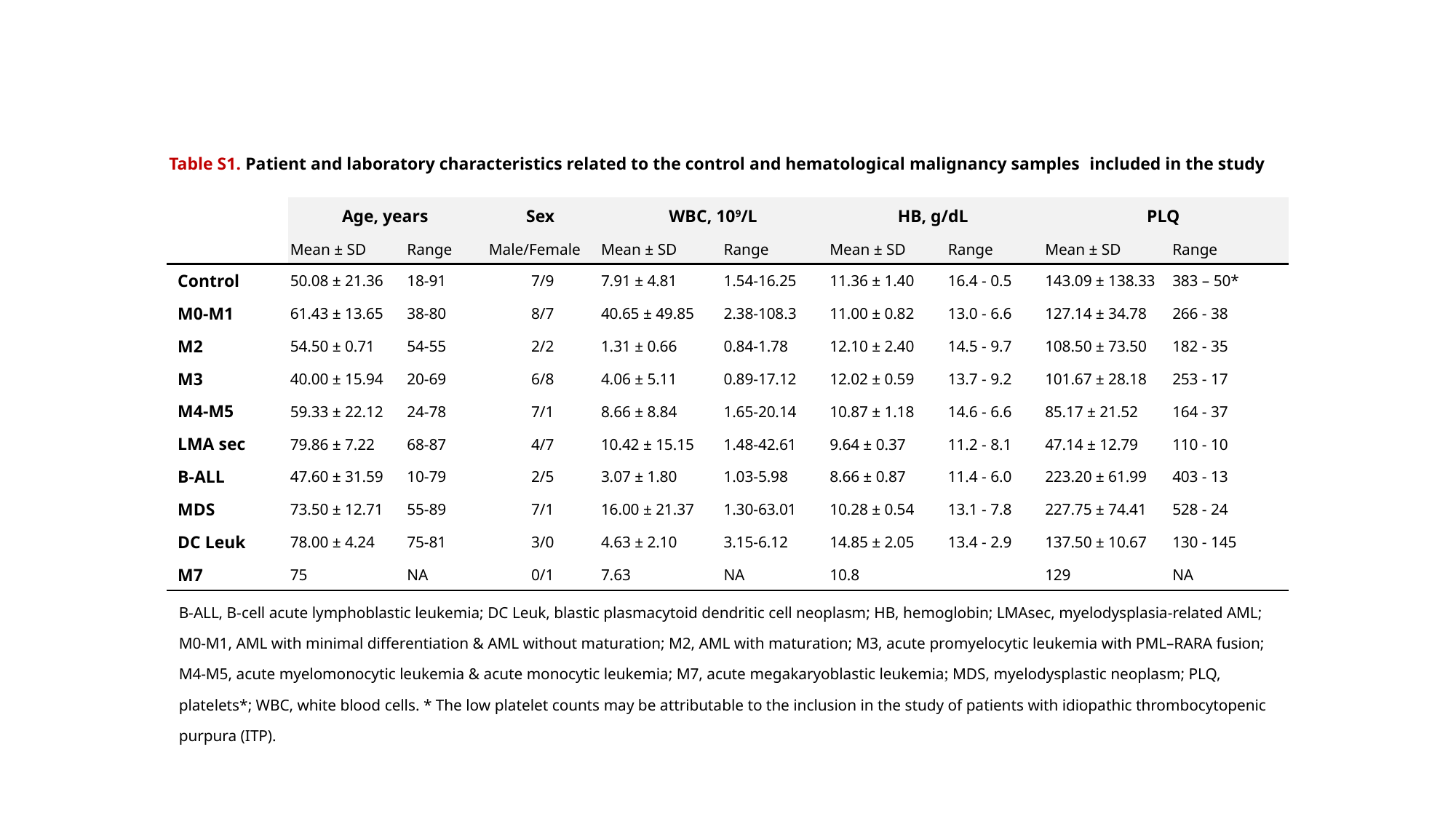

| Table S1. Patient and laboratory characteristics related to the control and hematological malignancy samples  included in the study | | | | | | | | | |
| --- | --- | --- | --- | --- | --- | --- | --- | --- | --- |
| | Age, years | | Sex | WBC, 109/L | | HB, g/dL | | PLQ | |
| | Mean ± SD | Range | Male/Female | Mean ± SD | Range | Mean ± SD | Range | Mean ± SD | Range |
| Control | 50.08 ± 21.36 | 18-91 | 7/9 | 7.91 ± 4.81 | 1.54-16.25 | 11.36 ± 1.40 | 16.4 - 0.5 | 143.09 ± 138.33 | 383 – 50\* |
| M0-M1 | 61.43 ± 13.65 | 38-80 | 8/7 | 40.65 ± 49.85 | 2.38-108.3 | 11.00 ± 0.82 | 13.0 - 6.6 | 127.14 ± 34.78 | 266 - 38 |
| M2 | 54.50 ± 0.71 | 54-55 | 2/2 | 1.31 ± 0.66 | 0.84-1.78 | 12.10 ± 2.40 | 14.5 - 9.7 | 108.50 ± 73.50 | 182 - 35 |
| M3 | 40.00 ± 15.94 | 20-69 | 6/8 | 4.06 ± 5.11 | 0.89-17.12 | 12.02 ± 0.59 | 13.7 - 9.2 | 101.67 ± 28.18 | 253 - 17 |
| M4-M5 | 59.33 ± 22.12 | 24-78 | 7/1 | 8.66 ± 8.84 | 1.65-20.14 | 10.87 ± 1.18 | 14.6 - 6.6 | 85.17 ± 21.52 | 164 - 37 |
| LMA sec | 79.86 ± 7.22 | 68-87 | 4/7 | 10.42 ± 15.15 | 1.48-42.61 | 9.64 ± 0.37 | 11.2 - 8.1 | 47.14 ± 12.79 | 110 - 10 |
| B-ALL | 47.60 ± 31.59 | 10-79 | 2/5 | 3.07 ± 1.80 | 1.03-5.98 | 8.66 ± 0.87 | 11.4 - 6.0 | 223.20 ± 61.99 | 403 - 13 |
| MDS | 73.50 ± 12.71 | 55-89 | 7/1 | 16.00 ± 21.37 | 1.30-63.01 | 10.28 ± 0.54 | 13.1 - 7.8 | 227.75 ± 74.41 | 528 - 24 |
| DC Leuk | 78.00 ± 4.24 | 75-81 | 3/0 | 4.63 ± 2.10 | 3.15-6.12 | 14.85 ± 2.05 | 13.4 - 2.9 | 137.50 ± 10.67 | 130 - 145 |
| M7 | 75 | NA | 0/1 | 7.63 | NA | 10.8 | | 129 | NA |
| B-ALL, B-cell acute lymphoblastic leukemia; DC Leuk, blastic plasmacytoid dendritic cell neoplasm; HB, hemoglobin; LMAsec, myelodysplasia-related AML; M0-M1, AML with minimal differentiation & AML without maturation; M2, AML with maturation; M3, acute promyelocytic leukemia with PML–RARA fusion; M4-M5, acute myelomonocytic leukemia & acute monocytic leukemia; M7, acute megakaryoblastic leukemia; MDS, myelodysplastic neoplasm; PLQ, platelets\*; WBC, white blood cells. \* The low platelet counts may be attributable to the inclusion in the study of patients with idiopathic thrombocytopenic purpura (ITP). | | | | | | | | | |

## Slide 3
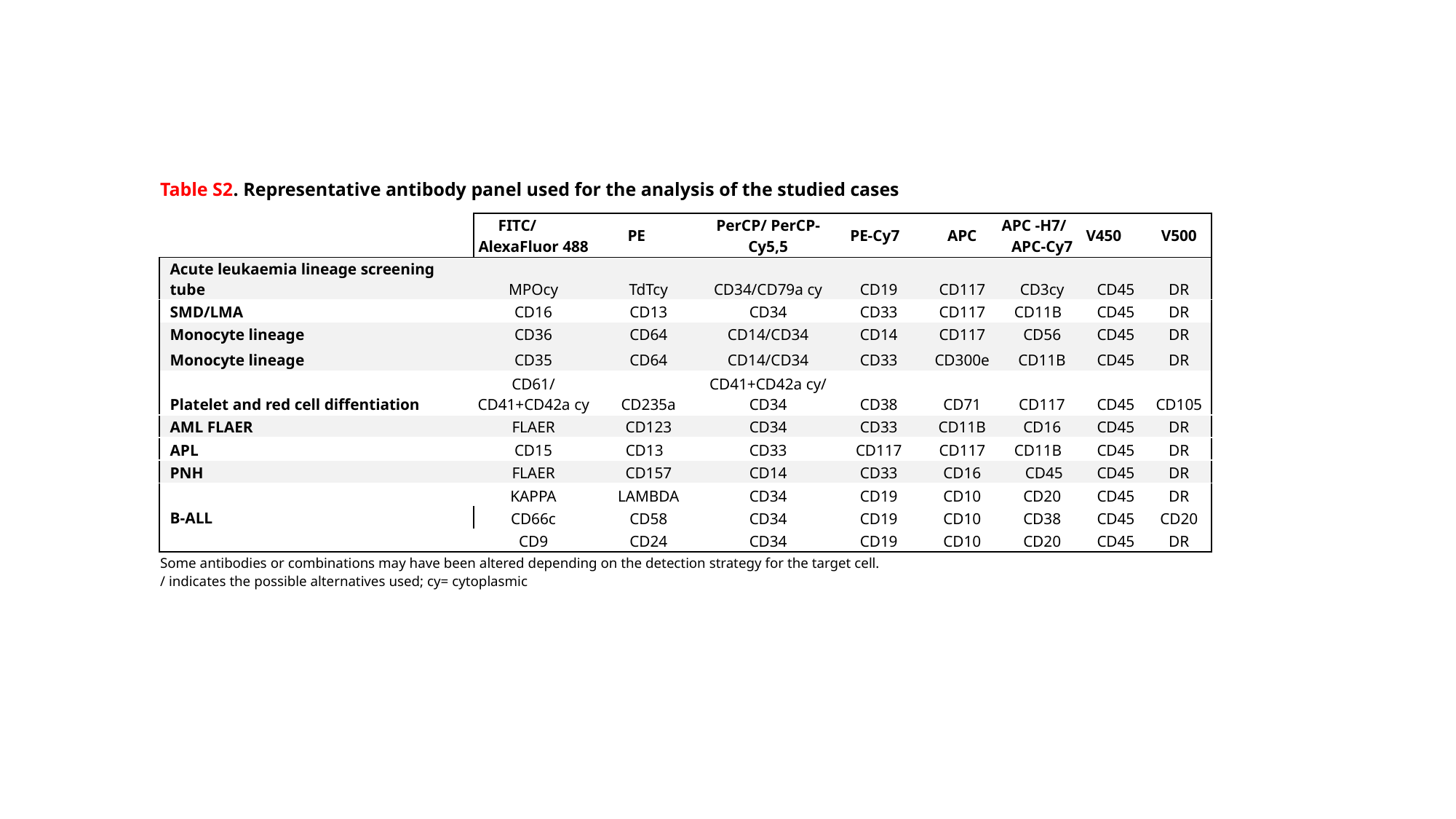

| Table S2. Representative antibody panel used for the analysis of the studied cases | | | | | | | | |
| --- | --- | --- | --- | --- | --- | --- | --- | --- |
| | FITC/ AlexaFluor 488 | PE | PerCP/ PerCP-Cy5,5 | PE-Cy7 | APC | APC -H7/ APC-Cy7 | V450 | V500 |
| Acute leukaemia lineage screening tube | MPOcy | TdTcy | CD34/CD79a cy | CD19 | CD117 | CD3cy | CD45 | DR |
| SMD/LMA | CD16 | CD13 | CD34 | CD33 | CD117 | CD11B | CD45 | DR |
| Monocyte lineage | CD36 | CD64 | CD14/CD34 | CD14 | CD117 | CD56 | CD45 | DR |
| Monocyte lineage | CD35 | CD64 | CD14/CD34 | CD33 | CD300e | CD11B | CD45 | DR |
| Platelet and red cell diffentiation | CD61/ CD41+CD42a cy | CD235a | CD41+CD42a cy/ CD34 | CD38 | CD71 | CD117 | CD45 | CD105 |
| AML FLAER | FLAER | CD123 | CD34 | CD33 | CD11B | CD16 | CD45 | DR |
| APL | CD15 | CD13 | CD33 | CD117 | CD117 | CD11B | CD45 | DR |
| PNH | FLAER | CD157 | CD14 | CD33 | CD16 | CD45 | CD45 | DR |
| B-ALL | KAPPA | LAMBDA | CD34 | CD19 | CD10 | CD20 | CD45 | DR |
| | CD66c | CD58 | CD34 | CD19 | CD10 | CD38 | CD45 | CD20 |
| | CD9 | CD24 | CD34 | CD19 | CD10 | CD20 | CD45 | DR |
| Some antibodies or combinations may have been altered depending on the detection strategy for the target cell. / indicates the possible alternatives used; cy= cytoplasmic | | | | | | | | |

## Slide 4
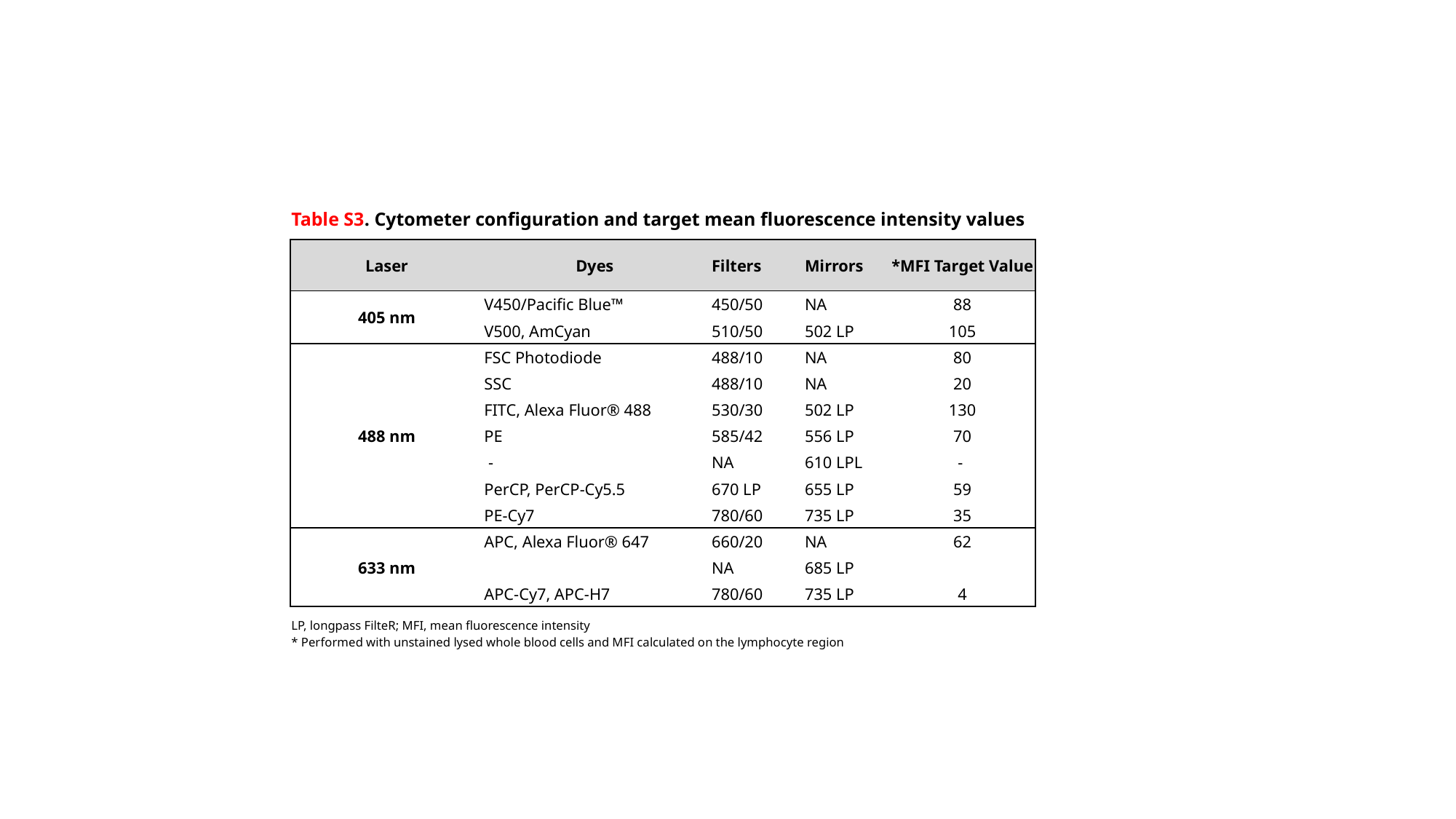

| Table S3. Cytometer configuration and target mean fluorescence intensity values | | | | |
| --- | --- | --- | --- | --- |
| Laser | Dyes | Filters | Mirrors | \*MFI Target Value |
| 405 nm | V450/Pacific Blue™ | 450/50 | NA | 88 |
| | V500, AmCyan | 510/50 | 502 LP | 105 |
| 488 nm | FSC Photodiode | 488/10 | NA | 80 |
| | SSC | 488/10 | NA | 20 |
| | FITC, Alexa Fluor® 488 | 530/30 | 502 LP | 130 |
| | PE | 585/42 | 556 LP | 70 |
| | - | NA | 610 LPL | - |
| | PerCP, PerCP-Cy5.5 | 670 LP | 655 LP | 59 |
| | PE-Cy7 | 780/60 | 735 LP | 35 |
| 633 nm | APC, Alexa Fluor® 647 | 660/20 | NA | 62 |
| | | NA | 685 LP | |
| | APC-Cy7, APC-H7 | 780/60 | 735 LP | 4 |
| LP, longpass FilteR; MFI, mean fluorescence intensity \* Performed with unstained lysed whole blood cells and MFI calculated on the lymphocyte region | | | | |

## Slide 5
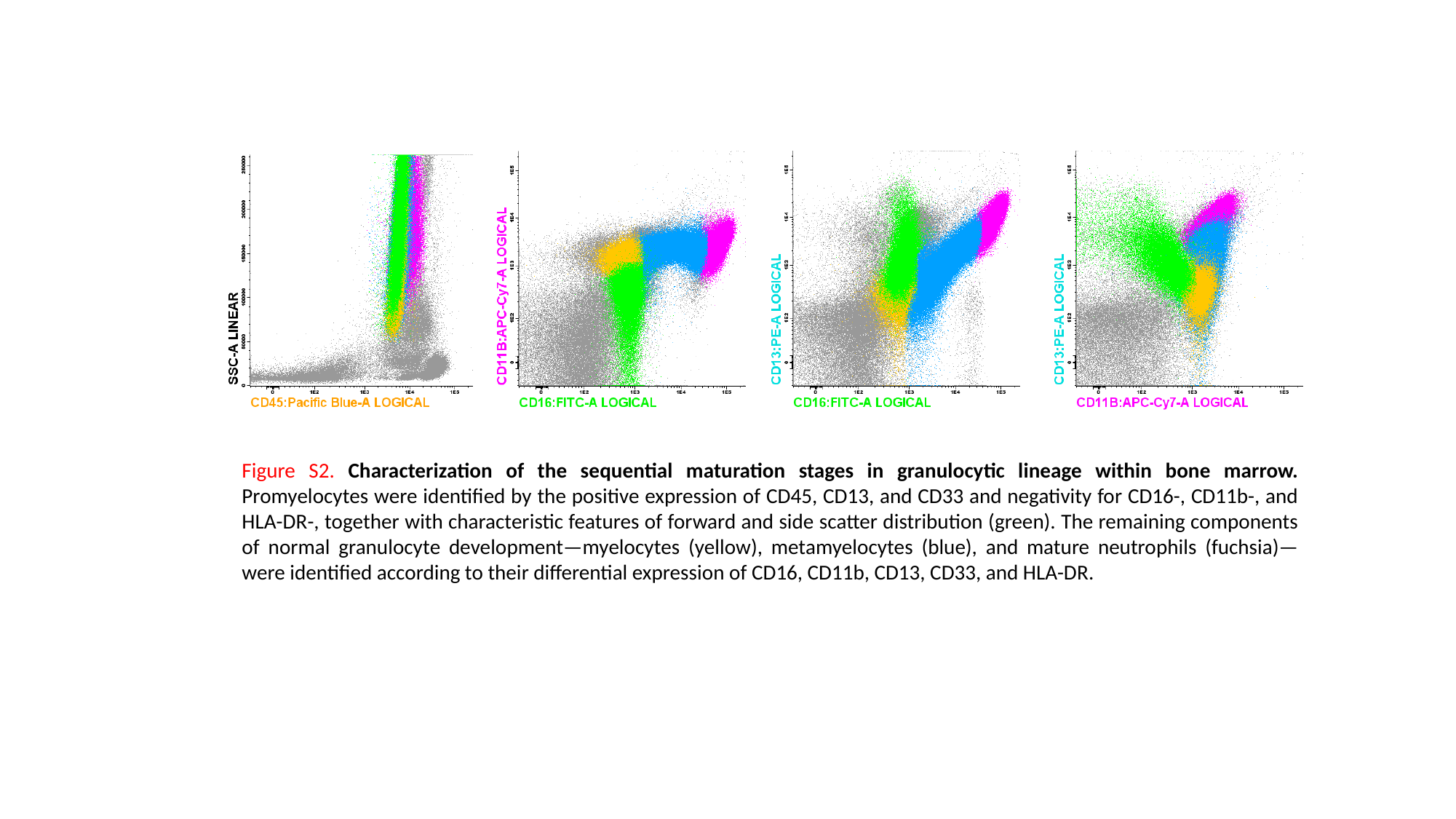

Figure S2. Characterization of the sequential maturation stages in granulocytic lineage within bone marrow. Promyelocytes were identified by the positive expression of CD45, CD13, and CD33 and negativity for CD16-, CD11b-, and HLA-DR-, together with characteristic features of forward and side scatter distribution (green). The remaining components of normal granulocyte development—myelocytes (yellow), metamyelocytes (blue), and mature neutrophils (fuchsia)—were identified according to their differential expression of CD16, CD11b, CD13, CD33, and HLA-DR.
